# Serum LncRNAs Profiles Serve as Novel Potential Biomarkers for the Diagnosis of HBV-Positive Hepatocellular Carcinoma

**DOI:** 10.1371/journal.pone.0144934

**Published:** 2015-12-16

**Authors:** Kang Wang, Wei xing Guo, Nan Li, Chun fang Gao, Jie Shi, Yu fu Tang, Feng Shen, Meng chao Wu, Shan rong Liu, Shu qun Cheng

**Affiliations:** 1 Eastern Hepatobiliary Surgery Hospital, The Second Military Medical University, Shanghai, China; 2 Changhai Hospital, the Second Military Medical University, Shanghai, China; University of Medicine, Greifswald, Germany, GERMANY

## Abstract

**Background:**

Hepatocellular carcinoma (HCC) is a common malignancy that has a poor prognosis because there is lack of methods for early diagnosis. We aimed to utilize two serum long non-coding RNAs (lncRNAs), uc001ncr and AX800134, to diagnose hepatitis B virus (HBV)–positive HCC.

**Methods:**

lncRNA microarrays were utilized to measure the differential expression of lncRNAs between tumor tissues and corresponding non-tumor tissues in HBV-positive hapatocellular carcinoma. uc001ncr and AX800134 were selected as candidate lncRNAs and detected in three independent cohorts containing a total of 684 participants (healthy individuals and chronic HBV patients and HBV-positive HCC patients) who were recruited between March 2011 and December 2012. A logistic regression model was constructed using a training cohort (n = 353) and validated using an independent cohort (n = 181). The area under the receiver operating characteristic curve (AUC) was utilized to evaluate the diagnostic accuracy.

**Results:**

We determined that a panel based on the expression of uc001ncr and AX800134 accurately diagnosed HBV-positive HCC (AUC values of 0.9494 and 0.9491 for the training and validation cohorts, respectively). The diagnostic performance of the panel remained high in patients with AFP≤400 ng/ml (AUC values of 0.9371 and 0.9527 for the training and validation cohorts, respectively). The panel also diagnosed early HCC (AUC values of 0.9450 and 0.9564 for the training and validation cohorts, respectively).

**Conclusion:**

Our results indicated that the serum expression of uc001ncr and AX800134 has potential as novel potential biomarker for the diagnosis of HCC, especially in patients with AFP≤400 ng/ml or early-stage disease (BCLC 0+A).

## Introduction

Hepatocellular carcinoma(HCC) is the sixth most common malignancy and has a 5-year overall survival rate of 5–9% [1–3]. The poor prognosis for this disease primarily results from late detection due to the lack of effective methods for early diagnosis [1, 4]. Assays for AFP, the traditional serum marker for HCC, are limited by low sensitivity and specificity [5–9]. Although other molecular markers have been identified for HCC, the heterogeneity of HCC makes early detection a major challenge.

Ideally, biomarkers should be accessible in specimens that can be collected conveniently, such as serum or urine. Highly stable cell-free circulating nucleic acids (cfCNAs), which include both RNA and DNA species, have been discovered in human blood, plasma, and urine [10]. Long non-coding RNAs (lncRNAs) are mRNA-like transcripts that are 200 bp to approximately 100 kb long, map to intronic and intergenic regions[11], and include subsets of polyadenylated and non-polyadenylated transcripts that differentially accumulate in the nucleus and cytoplasm of cells[12, 13]. While there is an increasing interest in lncRNAs, to date only a handful has been investigated in HCC, including highly up-regulated in HCC, such as HULC, HOTAIR, H19, HEIH and MVIH, and down-regulated in tumor tissues, such as MEG3, hDreh and LET. Those lncRNAs were identified to be significantly associated with tumorigenesis and metastasis in HCC patients. Published studies have suggested that lncRNAs have potential as biomarkers in human fluids; for example, compared with PSA (prostate-specific antigen) serum levels, the lncRNA PCA3 found in patient urine samples allowed for a more sensitive and specific diagnosis of prostate cancer [14–17]. The lncRNA HULC can be detected in the blood of HCC patients using conventional PCR methods and is highly expressed in tissue from HCC patients [18, 19]. However, the use of serum lncRNAs as early diagnostic markers for HCC has not been reported.

In China, chronic HBV infection is a major contributor to HCC[20]. This study hypothesized that the levels of specific circulating cancer-associated lncRNAs would differ between HBV-positive HCC patients, chronic HBV virus infected patients and healthy individuals. We measured lncRNA-uc001ncr and lncRNA-AX800134 in a cohort of 684 serum samples to identify a panel of lncRNAs that could diagnose HBV-positive HCC. The cohort included patients with chronic HBV,HBV-positive HCC patients, and healthy individuals.

## Materials and Methods

### Study Design and Patients

**Fig 1:**
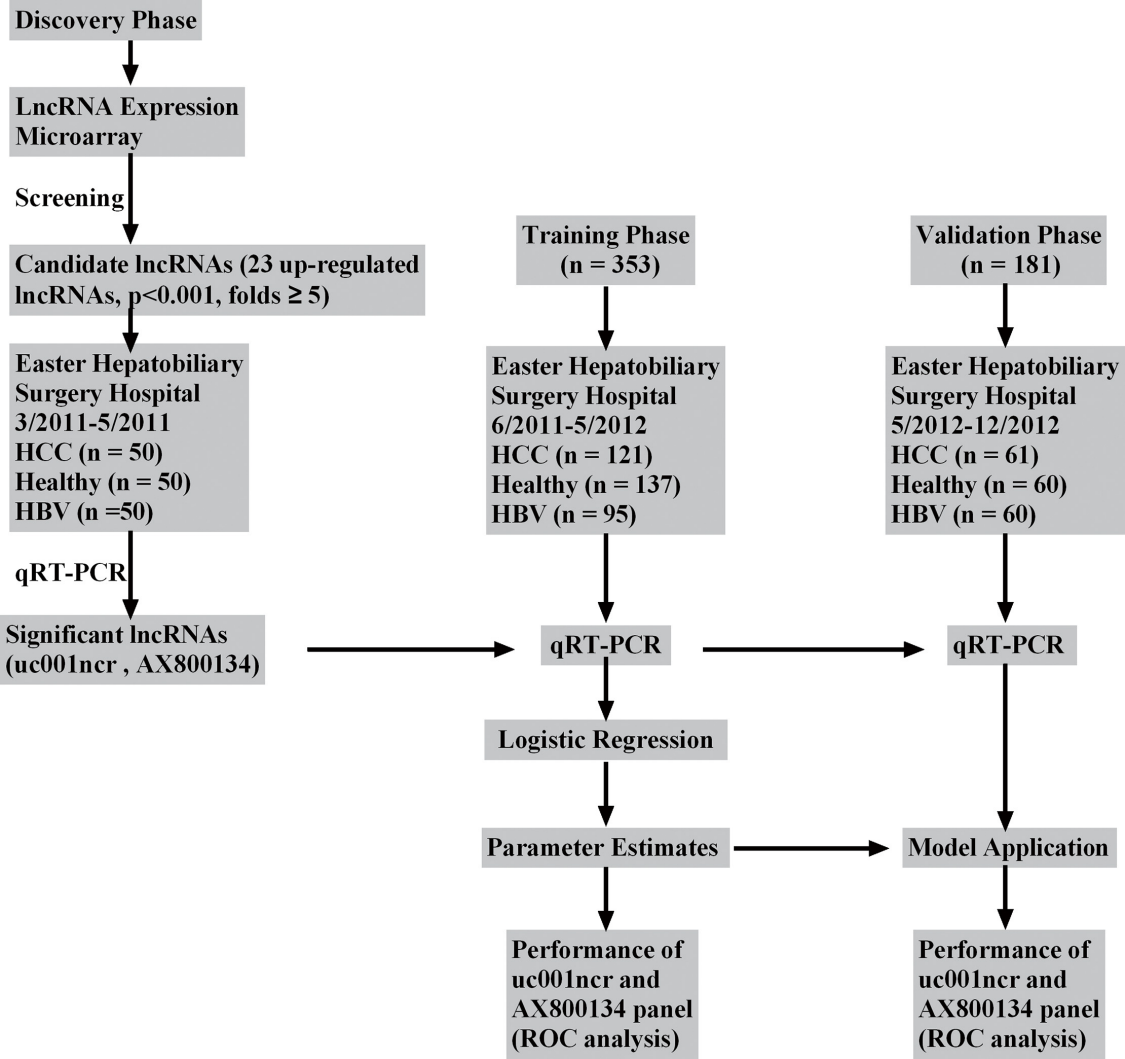
Study flow chart. HBV, chronic hepatitis B; HCC, hepatocellular carcinoma; ROC, receiver operating characteristics; qRT-PCR, Quantitative reverse transcription polymerase chain reaction.

Firstly, five HBV-positive HCC tumors and corresponding non-tumor liver samples were used to detect the differential expression of lncRNAs using a 12135K lncRNA Expression Microarray (ArrayStar,Rockville,MD). Secondly, 68 pairs of HCC and corresponding non-tumor liver tissue were used to validate the microarray analysis results). Finally, 684 blood samples including chronic HBV patients, HBV-positive HCC patients, and healthy individuals were separated into three phases in chronological order were utilized to validate the diagnostic value of the candidate lncRNAs for HCC patients. All the samples that met the eligibility criteria (S1 Table) were collected at the Eastern Hepatobiliary Surgery Hospital(EHSH) in Shanghai between Mar.2011 and Dec.2012. Written informed consent was obtained from all the patients. HCC was diagnosed histologically in the pathologic specimen. The study protocol was approved by the Institutional Ethics Committee (IEC) of the Eastern Hepatobiliary Surgery Hospital. Written informed consent was obtained from all the patients for their data to be used for research. The gene-specific primers used to detect lncRNAs are presented in S2 Table. Study flow chart was shown in Fig 1.

### Microarray and qRT-PCR

lncRNA microarrays were screened as previously described [21] using five HBV-positive HCC tissues and corresponding non-tumor liver samples (S3 Table). The p values were calculated using the paired t-test. The threshold for up- and down-regulated genes was a fold change ≥1.5 and a pvalue≤0.001. The microarray data discussed in this article have been deposited in National Center for Biotechnology Information (NCBI) Gene Expression Omnibus (GEO) and are accessible through (GEO) Series accession number GSE49713 (http://www.ncbi.nlm.nih.gov/geo/query/acc.cgi?acc=GSE49713). Hierarchical clustering was performed based on differentially expressed lncRNAs using Cluster and Tree View software from Stanford University (Palo Alto, CA). The details of the qRT-PCR assays are included in data in S1 Text.

### Validate the Microarray

To validate the microarray analysis results, we randomly quantitated 5 up-or down-regulated lncRNAs (S4 Table) identified in the microarray analysis by performing quantitative reverse transcription real-time polymerase chain reaction (qRT-PCR) on frozen tumor and corresponding non-tumor liver tissue from 68 Chinese patients (S5 Table cohort 1) with HBV-positive HCC who underwent a primary curative hepatectomy at EHSH between 2009 and 2010.

### Discovery Phase

Subsequently, 23 lncRNAs with significant p-value and larger expression fold changes (p<0.001 and an expression fold change≥5) in the lncRNA microarray were chosen on the basis of their potential relevance to HCC. After the measurement of 23 lncRNAs in 68 pairs of HCC and corresponding non-tumor liver tissue and a cohort of 150 serum samples including 50 HBV-positive HCC patients, 50 HBV patients and 50 healthy volunteers (S5 Table cohort 2). The uc001ncr and AX800134 were selected as candidate lncRNAs due to their significantly up-regulatation in both HCC tissues and serum samples compared with the control group with a detection rate of 100%. For the other 21 lncRNAs, the serum detection rates were <100%, or no significant differences were observed (S6 Table). Therefore, these lncRNAs were not included in further analytic studies.

### Training Phase

The two lncRNAs were analyzed by qRT-PCR in an independent cohort of serum samples from353 participants(121 HBV-positive HCCpatients,95 HBV patients and137 healthy individuals; Table 1). Data from these 353 participants were used to construct the diagnostic capability as the training set based on a logistic regression model to differentiate between the HCC(HBV patients) and the control groups.

**Table 1:** Characteristics of Study Participants in the Training and Validation Datasets.

	Training(n = 353)		Validation(n = 181)			
Variable	No.	%	No.	%		P
**HCC Count**	**121**		**61**			
Age (years)						0.3676
≤50	51	42.1	30	49.2		
> 50	70	57.9	31	50.8		
Sex						0.7486
Male	103	85.1	53	86.9		
Female	18	14.9	8	13.1		
Total bilirubin (mmol/l)						0.4458
≤18.8	99	81.8	47	77.0		
> 18.8	22	18.2	14	23.0		
ALT (U/l)						0.2534
≤44	78	64.5	34	55.7		
> 44	43	35.5	27	44.3		
Tumor size (cm)						0.7096
≤5	67	55.4	32	52.5		
> 5	54	44.6	29	47.5		
Tumor number						0.142
Single	93	76.9	55	90.2		
Multiple	28	23.1	9	9.8		
AFP(ng/ml)						0.4018
≤400	81	66.9	37			
> 400	40	33.1	24			
Macrovascular invasion						0.3116
Yes	49	40.5	20	32.8		
No	72	59.5	41	67.2		
cirrhosis						0.0213
Yes	77	63.6	49	80.3		
No	44	36.4	12	19.7		
BCLC						0.7167
0+A	70	57.9	37	60.7		
B+C	51	42.1	24	39.3		
HbsAg						NA
Positive	121	100.0	61	100.0		
**HBV Count**	**95**		**60**			
Age (years)						0.5111
≤50	77	81.1	46	76.7		
> 50	18	18.9	14	23.3		
Sex						0.9321
Male	50	52.6	32	53.3		
Female	45	47.4	28	46.7		
Total bilirubin (mmol/l)						0.7382
≤18.8	75	78.9	46	76.7		
> 18.8	20	21.1	14	23.3		
ALT (U/l)						< 0.001
≤44	83	87.4	22	36.7		
> 44	12	12.6	38	63.3		
AFP(ng/ml)						NA
≤400	95	100.0	60	100.0		
**Healthy Count**	**137**		**60**			
Age (years)						0.5747
≤50	63	45.7	24	40.0		
> 50	74	53.6	36	60.0		
Sex						0.4726
Male	112	81.8	55	90.2		
Female	15	18.2	5	8.3		
Total bilirubin (mmol/l)						0.4838
≤18.8	121	88.3	55	91.7		
> 18.8	16	11.7	5	8.3		
ALT (U/l)						0.5871
≤44	135	98.5	58	96.7		
> 44	2	1.5	2	3.3		
AFP(ng/ml)						NA
≤400	137	100.0	60	100.0		

Abbreviations: AFP, alpha fetoprotein; BCLC, Barcelona Clinic Liver Cancer; ALT, alanine aminotransferase; HCC, hepatocellular carcinoma; HBV, chronic hepatitis B; %, the percentage of the total.

NA, not application.

### Validation Phase

We utilized another independent cohort of serum samples from 61 HBV-positive HCC patients, 60 HBV patients and 60 healthy individuals (Table 1) to validate the diagnostic performance of the two lncRNAs based on the parameters in the logistic model from the training phase.

### Statistical Analyses

For the qRT-PCR data, the unpaired or paired t-test was used to compare the HBV positive HCC and control groups. A logistic regression model was applied to select diagnostic lncRNA markers based on the training cohort[22, 23]. A receiver operating characteristic (ROC) curve was constructed and the area under the ROC curve (AUC) was used to evaluate the diagnostic performance of the selected lncRNA panel[24]. The analyses were performed with the SPSS for Windows 20.

## Results

### Patient Characteristics

The clinical characteristics of the participants for the training and validation cohorts are shown in Table 1. There was no significant differences in the age and sex distribution between the training and validation datasets for the three groups (healthy people, HBV patients, and HBV-positive HCC patients). In the HBV-positive HCC group, there were fewer participants with cirrhosis in the training cohort than in the validation cohort(63.64% vs. 80.33%; P = 0.027). In the HBV group, the number of patients with alanine aminotransferase (ALT)>44u/l was significantly different between the two cohorts(12.63% vs. 63.33% for the training and validation cohorts, respectively; P<0.001). The other characteristics of the participants were similar in the two cohorts.

### LncRNA Screening and the Microarray Validation

**Fig 2:**
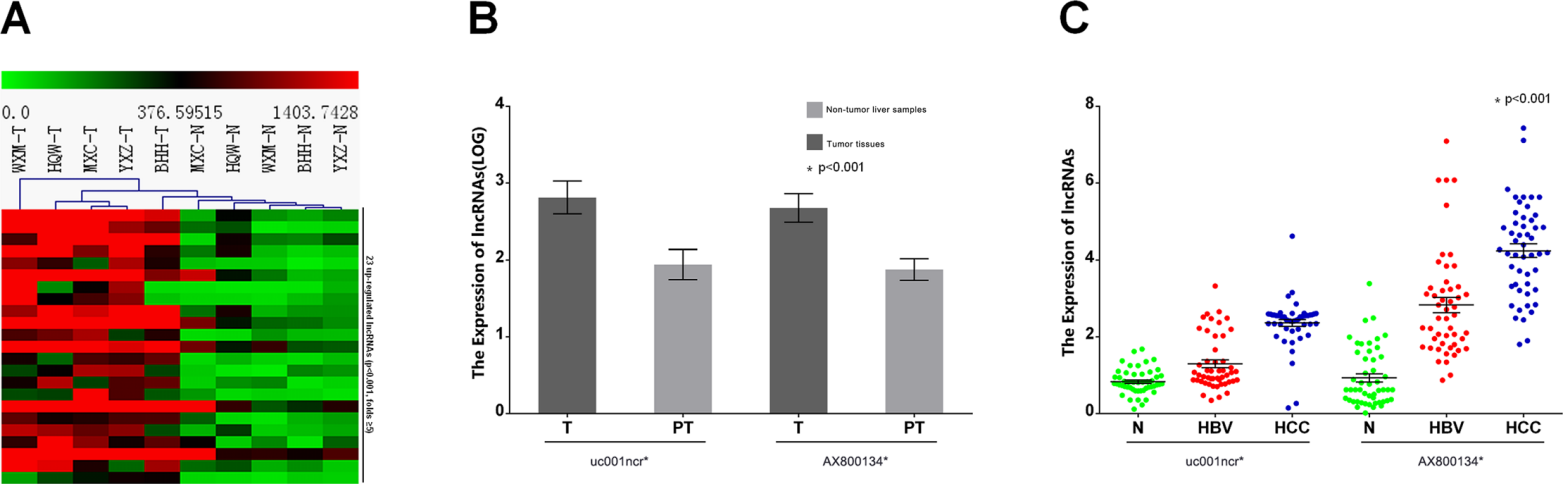
Differential expression of lncRNAs in HCC. (A)Hierarchical clustering analysis of 23lncRNAs that were up regulated in the HCC (T, tumor tissue) and non-tumor (N, paired non-tumor tissue) samples (fold change ≥5.0; p< 0.001). The lncRNA expression levels are represented in shades of red and green, indicating expression above and below the median expression value for all the samples.(B) The expression levels of uc001ncr and AX800134 in 68 HCC tumor tissue (T) and paired non-tumor liver samples (PT) (* p<0.001). (C) The expression levels of uc001ncr and AX800134 in 50 HCC and control serum samples (* p<0.001; control, HBV patients and healthy volunteers; N, healthy volunteers).

There were 23 up-regulated lncRNAs with p<0.001 and an expression fold change≥5 among 1276 up-regulated lncRNAs with P<0.001 and an expression fold change≥1.5 in HBV-positive HCC tumor tissue compared with corresponding non-tumor liver tissue (Fig 2A). To validate the microarray analysis, we randomly selected five lncRNAs (S4 Table) from the differentially expressed lncRNAs and analyzed their expression by qRT-PCR in 68 pairs of HCC and corresponding non-tumor liver tissue (S5 Table, Cohort 1). The data confirmed that AK128595, AX800134and uc001ncr had significantly higher expression levels in tumor tissue than in corresponding non-tumor liver tissue, whereas uc009ycz and NR_027300 were significantly down-regulated (P<0.001; S1 Fig).

### Differential Expression of 23 Up-Regulated lncRNAs in the HBV-Positive HCC and Control Groups

23 up-regulated lncRNAs with P<0.001 and an expression fold change≥5 in HBV-positive HCC tumor tissue compared with corresponding non-tumor liver tissue were identified and analyzed by qRT-PCR in 68 pairs of HCC and corresponding non-tumor liver tissue and 150 serum samples (50 healthy volunteers, 50 HBV patients and 50 HBV-positive HCC patients; S5 Table, Cohort 2). The results revealed a 100% detection rate for uc001ncr and AX800134 and a significant difference (p<0.001) between the HBV-positive HCC, HBV patients and healthy volunteers serum samples (Fig 2B and 2C), whereas 21 of the 23lncRNAshad a detection rate of<100% or exhibited no significant difference in expression between the HBV-positive HCC serum samples and the control samples (S6 Table). Therefore, uc001ncr and AX800134 were identified as candidates for additional qRT-PCR testing.

### The Expression Profile of Two LncRNAs in the Training Dataset

**Fig 3:**
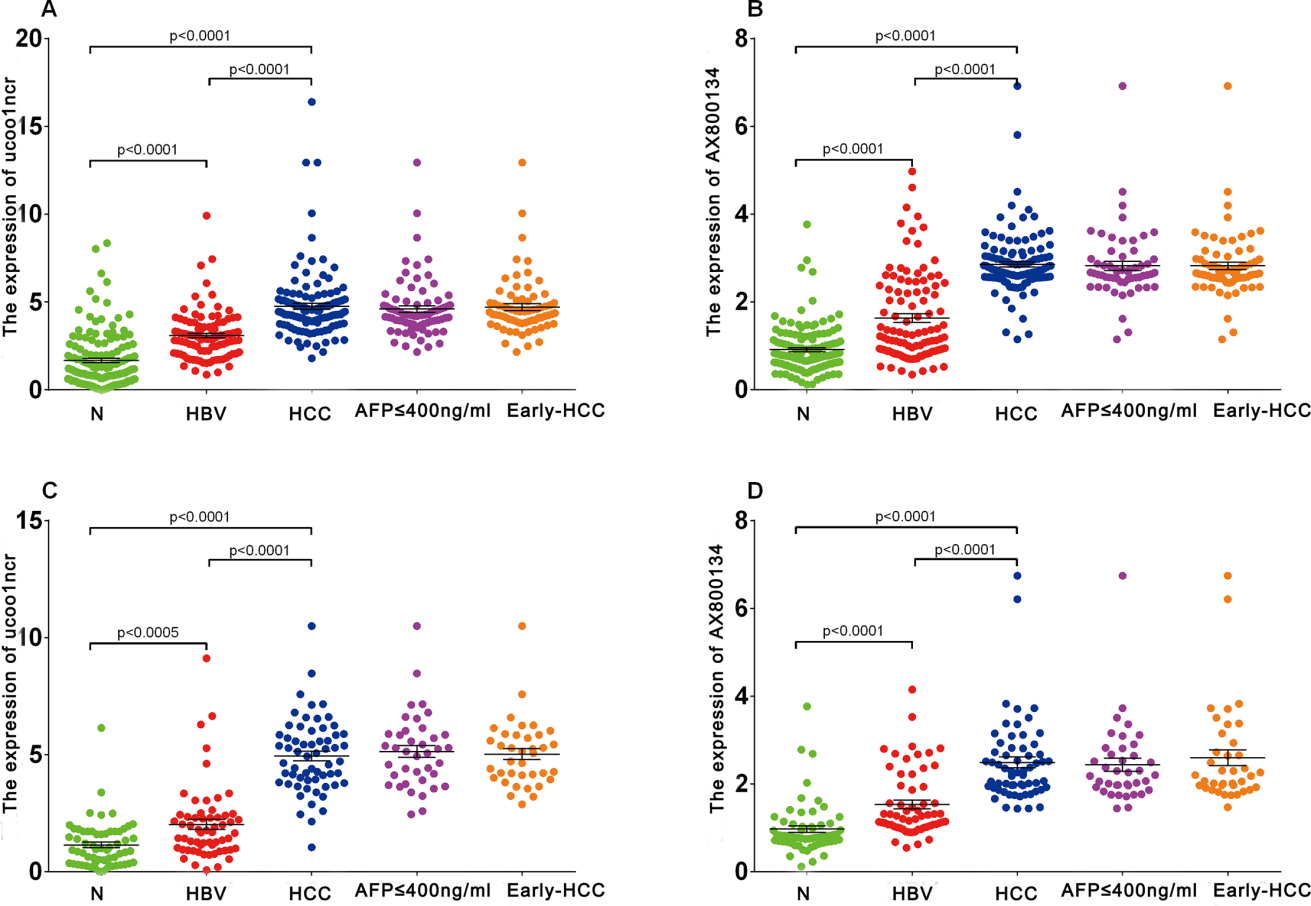
The serum expression levels ofuc001ncr and AX800134 in the training and validation cohorts. (A) uc001ncr, training cohort. (B) AX800134, training cohort. (C) uc001ncr, validation cohort. (D) AX800134, validation cohort. Black horizontal lines represent the mean, and the error bars are calculated as the SE. N, healthy volunteers; HBV, chronic hepatitis B virus infection; HCC, hepatocellular carcinoma; AFP, α-fetoprotein.

The serum expression of uc001ncr in HBV positive patients was significantly higher than HBV patients and healthy volunteers in training dataset(1.54 fold for HBV patients and 2.83 fold for healthy volunteers, p<0.001). Similar results was observed for AX800134 (1.89 fold for HBV patients and 3.32 fold for healthy volunteers, p<0.001, Fig 3A and 3B).

### Establishing the Predictive lncRNA Panel in the HBV- Positive HCC Group and Control Group

We set the HBV patients and healthy volunteers as control group. Increased expression of uc001ncr and AX800134 was observed in the HBV-positive HCC group compared with the control group (2.08-fold and 2.34-fold for uc001ncr and AX800134, respectively; Table 2, Fig 3A and 3B). The corresponding AUCs were 0.8859 for uc001ncr and 0.9251 for AX800134 (S2A and S2B Fig). The multivariate p values for the two lncRNAs were<0.0001 (Table 2).

**Table 2:** lncRNA Profile and Diagnostic Performance in Training Dataset.

	HCC Versus Control			
	Univariate			Multivariate
lncRNA	P	Fold Change	AUC	P
uc001ncr	<0.0001	2.08	0.8859	<0.0001
AX800134	<0.0001	2.34	0.9251	<0.0001

Note. lncRNA panel AUC = 0.9494 (95% CI, 0.9274 to 0.9721).

Abbreviations: AUC, area under the receiver operating characteristic curve;

HCC, hepatocellular carcinoma.

Control group includes healthy participants, patients with chronic hepatitisB(HBV).

Logit (P = HCC) = -7.531+0.763*uc001ncr +1.976* AX800134.

**Fig 4:**
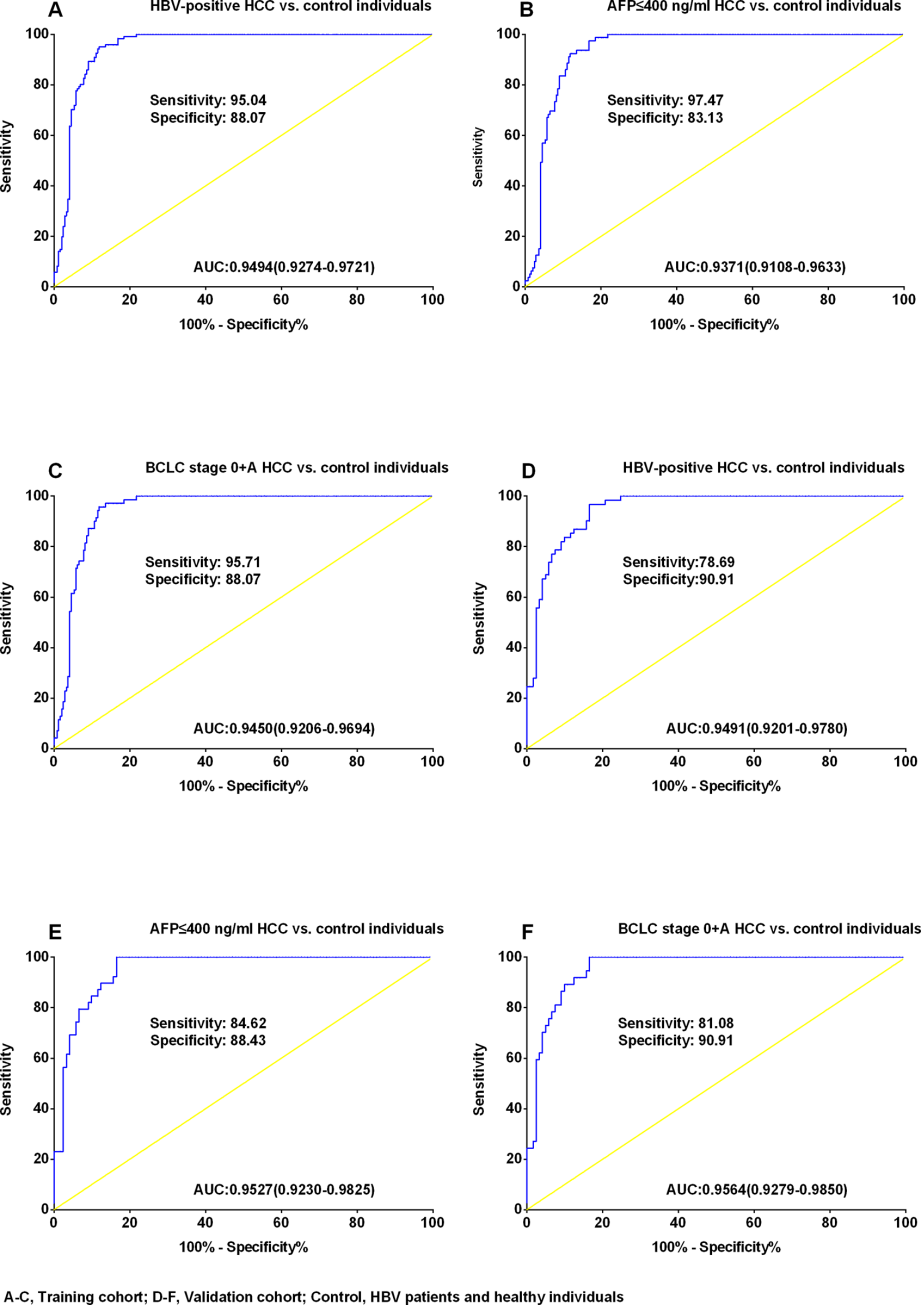
Receiver operating characteristic curve analysis for diagnosing hepatocellular carcinoma. Areas under the curve (AUCs) estimated for the uc001ncr and AX800134 panel in the training cohort (A-C) and the validation cohort(D-F), (A)HBV-positive HCC patients versus(vs.) control individuals, (B) the AFP≤400ng/ml samples vs. control individuals, (C) the Barcelona Clinic Liver Cancer (BCLC) stage 0+Asamples vs. control individuals, (D) HBV-positive HCC patients versus(vs.) control individuals, (E)the AFP≤400 ng/ml samples vs. control individuals, (F)the BCLC stage 0+Asamples vs. control individuals. Control, HBV patients and healthy volunteers; N, healthy volunteers. The optimum predicted probabilities of lncRNA-panel was derived from their respective ROC curves by maximizing the sum of sensitivity and specificity and minimizing the overall error [square root of the sum (1-sensitivity)^2^+ (1-specificity)^2^ as well as minimizing the distance of the cutoff value to the top-left corner of the ROC curve.

To estimate the probability of being diagnosed with HCC in the control group, a logistic regression model was constructed to apply to the training set (353 samples) using parameters based on the two significant lncRNAs. The following equation was used to construct the ROC curve (Table 2): Logit(P = HCC) = -7.531+0.763*uc001ncr +1.976*AX800134. The AUC for the two lncRNAs in discriminating HBV-positive HCC patients from control individuals was 0.9494(95% CI: 0.9274–0.9721; Fig 4A). At the optimal cut-off value of 0.3676, the sensitivity and specificity for this marker was 95.04% and 88.07%. The AUC for the two lncRNAs in discriminating the AFP≤400 ng/ml, HBV-positive HCC group from the control group was 0.9371 (95% CI: 0.9108–0.9633; Fig 4B). At the optimal cut-off value of 0.2430, the sensitivity and specificity for this marker was 97.47% and 83.13%.The AUC for the two lncRNAs in discriminating early HBV-positive HCC(BCLC 0+A stage) from control was 0.9450 (95% CI: 0.9206–0.9694; Fig 4C). At the optimal cut-off value of 0.3676, the sensitivity and specificity for this marker was 95.71% and 88.07%.

### Validating the Two LncRNAs

The expression levels of the two lncRNAs were determined in an independent validation cohort (181 serum samples; Fig 3C and 3D, S2C and S2D Fig).Using the parameters estimated based on the training cohort, the probability of being diagnosed with HCC was calculated in the validation cohort, and ROC curves were generated. The AUC was 0.9491 (95% CI: 0.9201–0.9780; Fig 4D) for discriminating HBV-positive HCC from control, 0.9527 (95% CI: 0.9230–0.9825; Fig 4E) for discriminating AFP≤400 ng/ml, HBV-positive HCC from control, and 0.9564(95% CI: 0.9279–0.9850; Fig 4F) for discriminating early HBV-positive HCC(BCLC 0+A stage) from control. At the corresponding optimal cut-off of training cohort, the sensitivity and specificity were 78.69% and 90.91%., 84.62% and 88.43%. and 81.08% and90.91% for discriminating HBV-positive HCC from control, AFP≤400 ng/ml, HBV-positive HCC from control and early HBV-positive HCC (BCLC 0+A stage) from control, respectively.

## Discussion

HCC has an extremely poor prognosis and is one of the most common and aggressive human malignancies worldwide. The imaging and biomarker tests are the principal methods for diagnosing HCC at present. However, for early-stage HCC, these methods are unsatisfactory. Our findings suggest the potential of utilizing circulating lncRNAs as non-invasive serological biomarkers for HCC as previously report for HULC.

Recently, lncRNAs have been identified that have altered expression in various types of human cancer[25, 26]. We have identified non overlapping signatures of a few lncRNAs that are aberrantly expressed in human HBV-positive HCC compared with paired non-tumor liver tissue. These lncRNAs may play tumor suppressor or oncogenic roles[27–29], similar to MALAT1, NEAT1, AOC4P and HOTAIR[21, 30, 31]. According to published papers, MALAT-1 may regulate alternate splicing by modulating the activity of serine/arginine (SR) splicing factors that regulate alternative splicing [31]. High NEAT1 expression levels acts as a pivotal player in tumorigenesis and metastasis of HCC[32], LncRNA AOC4P is a tumor suppressor for HCC by enhancing vimentin degradation and suppressing the epithelial mesenchymal transition(EMT)[33]. HOTAIR silence activates P16^Ink4a^ and P14^ARF^ signaling by enhancing miR-218 expression and suppressing Bmi-1 expression, which suppressed the tumorigenesis in HCC[34].

Until now, only a few circulating lncRNAs (for example, PCA3 and HULC) have been identified and have been determined to be good tumor diagnostic markers. Serum lncRNAs, SPRY4-IT1, ANRIL, NEAT1, XIST and HIF1A-AS1 were identified as the potential predictor for the tumorigenesis of non-small-cell lung cancer[35, 36]. Lei Dong etc. reported serum lncRNAs: CUDR, LSINCT-5 and PTENP1 were identified as diagnostic marker for gastric cancer[37]. lncRNA RP11-445H22.4 was reported may be a new potential biomarker of breast cancer[38]. It has been hypothesized that the presence of cfCNAs is related to the apoptosis and necrosis of cancer cells in the tumor microenvironment oris the result of secretion[39, 40]. The strong correlation between tumor-associated changes in genomic, epigenetic, or transcriptional patterns and alterations in cfCNAs levels strongly suggest the potential of these biomarkers as clinical tools[41]. Our results indicated that serum lncRNAs might be effective at diagnosing HCC. Circulating cfCNAs, such as miRNAs, can be detected in the serum and plasma of cancer patients because they are surprisingly stable despite the high levels of RNases that circulate in the blood. This implies that miRNAs might be protected from degradation by packaging into micro particles, such asexosomes, micro vesicles, apoptotic bodies and apoptotic micro particles[40]. Thus far, the reported RNA content of micro vesicles and exosomes includes primarily small miRNAs and long protein-coding mRNAs[42]. Like miRNAs, lncRNAs, which are 200 nt to approximately 100 kb long, are also packaged into micro particles, only B22 nt long? Which need further investigation.

Limited information of the two lncRNAs is available in HBV and liver cirrhosis, and its molecular mechanisms and roles in pathogenesis remain largely unknown. Huang, etc. [43] reported lncRNA down-regulated expression by HBx (lncRNA-Dreh) was not the same between hepatitis B virus X protein (HBx) transgenic mice and wild type mice and it can supress HCC growth and metastasis in vitro and in vivo through cytoskeletalmodulation by repressed expression of vimentin. In our study, the expression level of two lncRNA was up-regulated in HBV patients compared to healthy people, could this alter be also associated with HBx? Which need further study. By comparing the expression level of two lncRNAs between the HCC patients with liver cirrhosis and the patients without liver cirrhosis (S3A Fig), and HCC patients with normal or abnormal ALT levels S3B Fig), there wasn’t significant difference was found in both training cohort and validation cohort, this may imply that a potential liver cirrhosis and ALT levels couldn’t effect on lncRNAs expression levels.

For many years, AFP has been used for diagnosing and screening for HCC [44, 45]. However, It's poor sensitivity for detecting HCC and increase in the absence of HCC (such as in cases of chronic hepatitis or cirrhosis) has prompted the search for novel markers of HCC[46, 47]. There was approximately30-40% of HCC patients with low AFP levels basing on a cutoff of 400 ng/ml is typically used [41, 42]. We have demonstrated that the serum expression level of two lncRNAs was not related to AFP levels (S4 Fig) that may suggested that the two lncRNAs levels in HCC patients are not related to liver regeneration or necrosis, these two lncRNAs could be a more sensitive marker for early HCC or AFP<400ng/ml HCC than AFP is, which may be useful for the tumor respectability at diagnosis. We used BCLC stage 0+A to define early-stage HCC[48].

This study failed to identify the prognosis for the HCC patients included in our study. Global profiling of circulating lncRNAs has yet to be performed because current limited technologies made it difficult to perform the lncRNA microarrays using serum samples. These results highlight the need to validate the microarray data using more accurate and complementary techniques.

Our results indicated that serum lncRNAs have potential as novel potential biomarkers for the diagnosis of HCC, especially for patients with AFP≤400 ng/ml or early-stage disease (BCLC 0+A).

## Supporting Information

S1 Fig Five randomly quantitated 5 up- or down-regulated lncRNAs in 68 paired HCC and non-tumor liver samples using qRT-PCR (*p<0.001). (TIF)

S2 Fig Receiver operating characteristic curve analysis for diagnosing hepatocellular carcinoma. Area under the curve (AUC) estimate for (A)uc001ncr in the training cohort, (B) AX800134 in the training cohort, (C) uc001ncr in the validation cohort, and (D) AX800134 in the validation cohort.
(TIF)

S3 Fig The serum expression levels of uc001ncr and AX800134 between HCC in patients with cirrhosis versus HCC in patients without cirrhosis and normal ALT level or abnormal ALT level. (A) Patients with normal ALT and abnormal ALT level, (B) Patients with or without liver cirrhosis.
(TIF)

S4 Fig The The correlation between uc001ncr and AX800134 level and AFP level. A for uc001ncr and B for AX800134.
(TIF)

S1 Table Eligibility Criteria for Selection of the Subjects. (DOCX)

S2 Table Primary Oligonucleotide Sequences used in this study. (DOCX)

S3 Table Clinical characteristics of 5 HCC patients used for lncRNA. (DOCX)

S4 Table Expression Profiles of 5 lncRNAs on Microarrays. (DOCX)

S5 Table Clinical Characteristics of the HCC Patients. (DOCX)

S6 Table Expression Profiles of 22 Candidate lncRNAs on qRT-PCR in 150 Samples. (DOCX)

S1 Text The details of the qRT-PCR. (DOCX)

## Abbreviations

(HCC): Hepatocellular carcinoma
(lncRNAs): long non-coding RNAs
(HBV): hepatitis B virus
(AUC): The area under the receiver operating characteristic curve
(cfCNAs): cell-free circulating nucleic acids
(NCBI): Biotechnology Information
(GEO): Gene Expression Omnibus
(qRT-PCR): quantitative reverse transcription real-time polymerase chain reaction
(ROC): receiver operating characteristic
(ALT): alanine aminotransferase
