# Supplementary figures and images for: Serum LncRNAs Profiles Serve as Novel Potential Biomarkers for the Diagnosis of HBV-Positive Hepatocellular Carcinoma
Source: PLoS One. 2015 Dec 16;10(12):e0144934. doi: 10.1371/journal.pone.0144934 (PMC4684503; doi:10.1371/journal.pone.0144934)

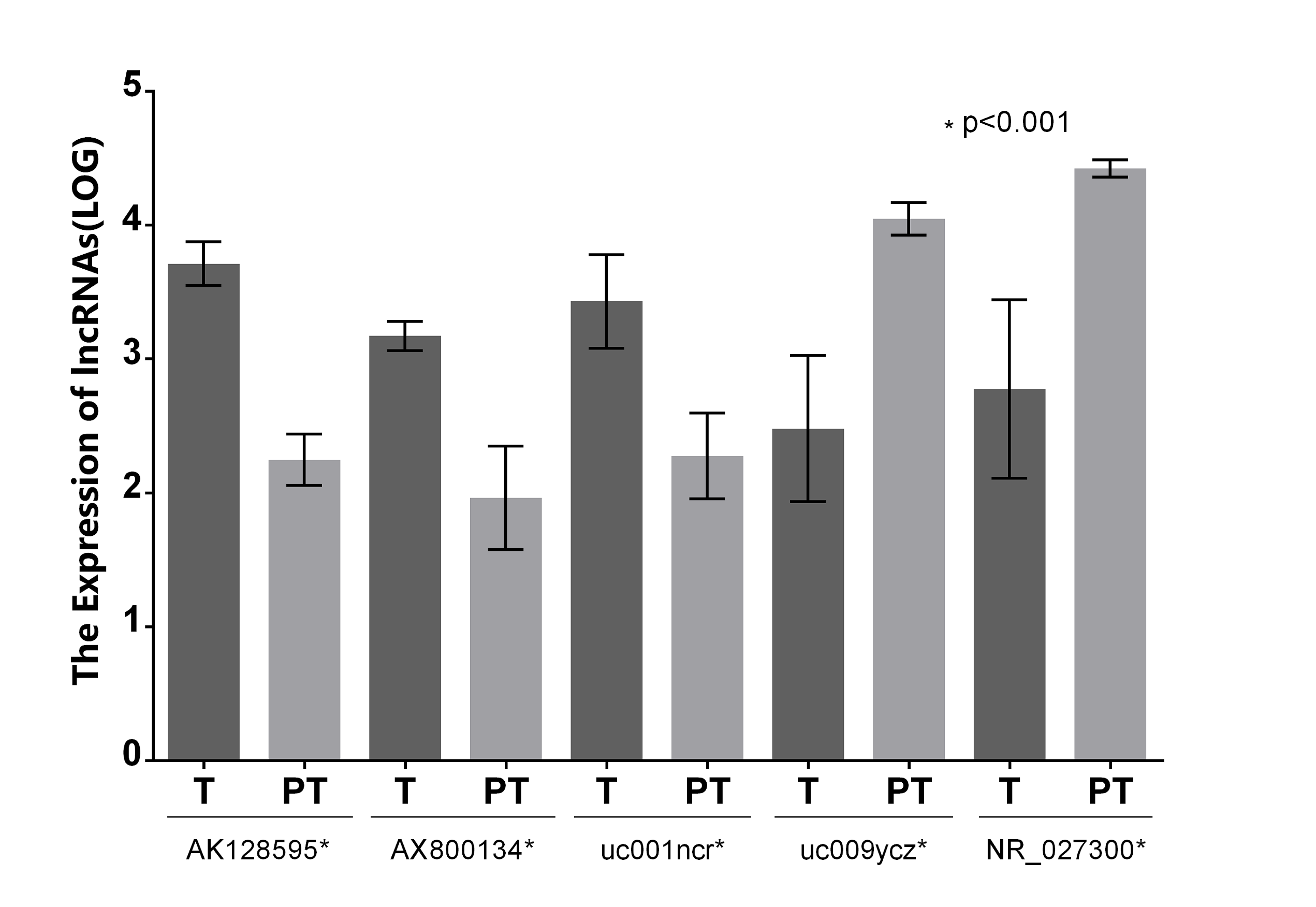

Supplement: S1 Fig — (TIF) [file pone.0144934.s001.tif]

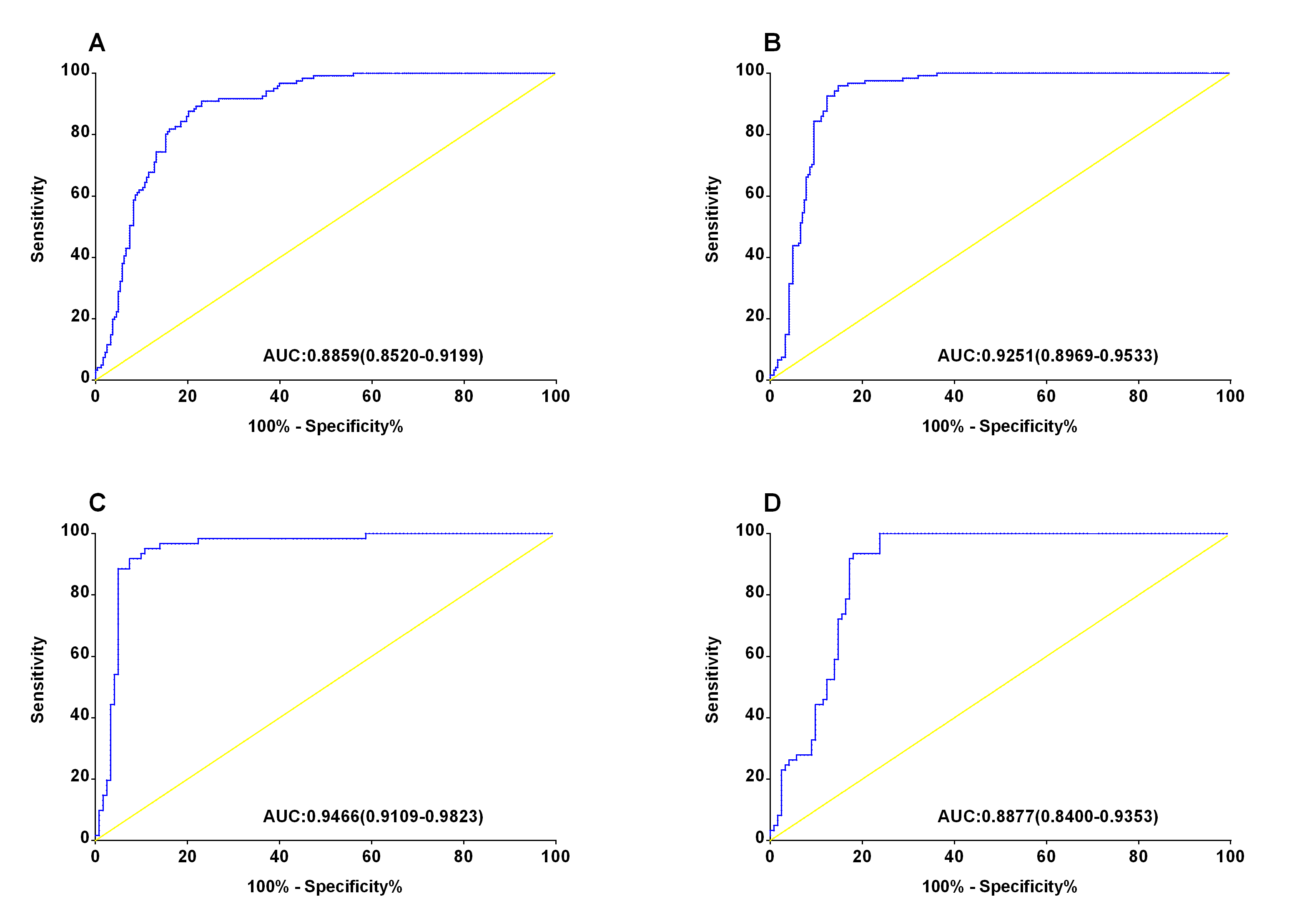

Supplement: S2 Fig — Area under the curve (AUC) estimate for (A)uc001ncr in the training cohort, (B) AX800134 in the training cohort, (C) uc001ncr in the validation cohort, and (D) AX800134 in the validation cohort. (TIF) [file pone.0144934.s002.tif]

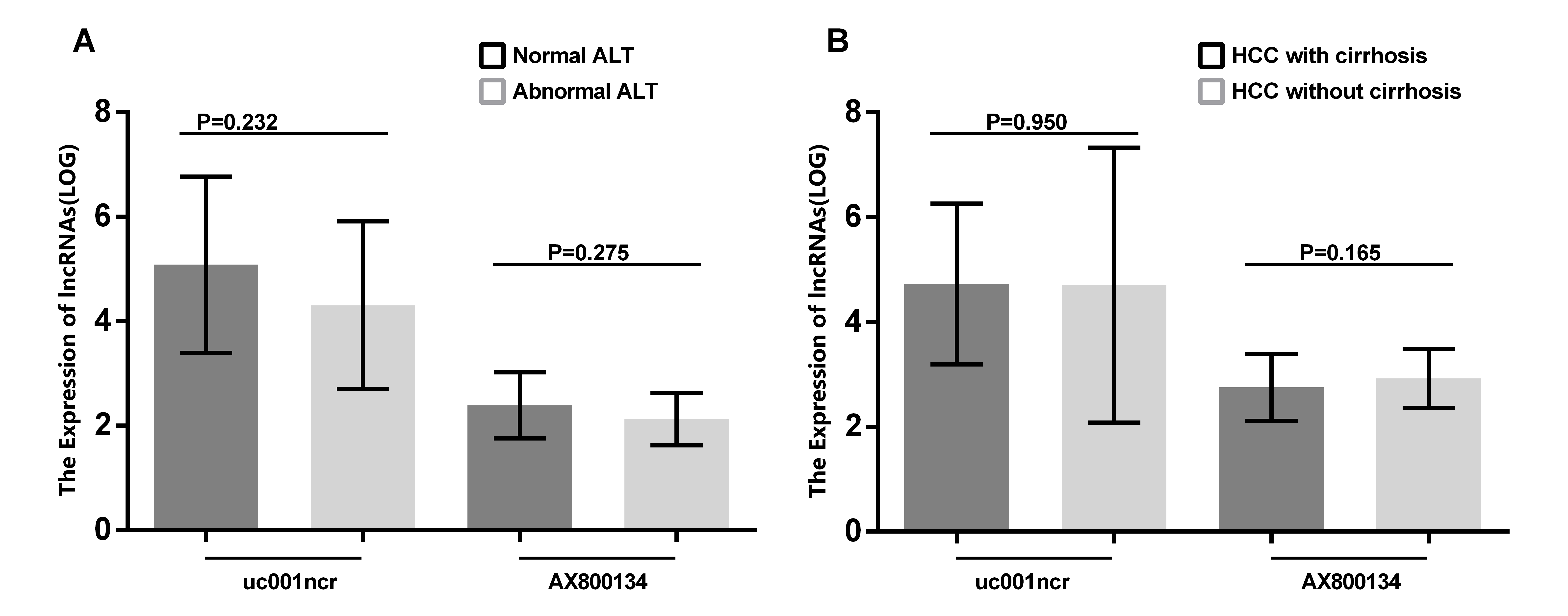

Supplement: S3 Fig — (A) Patients with normal ALT and abnormal ALT level, (B) Patients with or without liver cirrhosis. (TIF) [file pone.0144934.s003.tif]

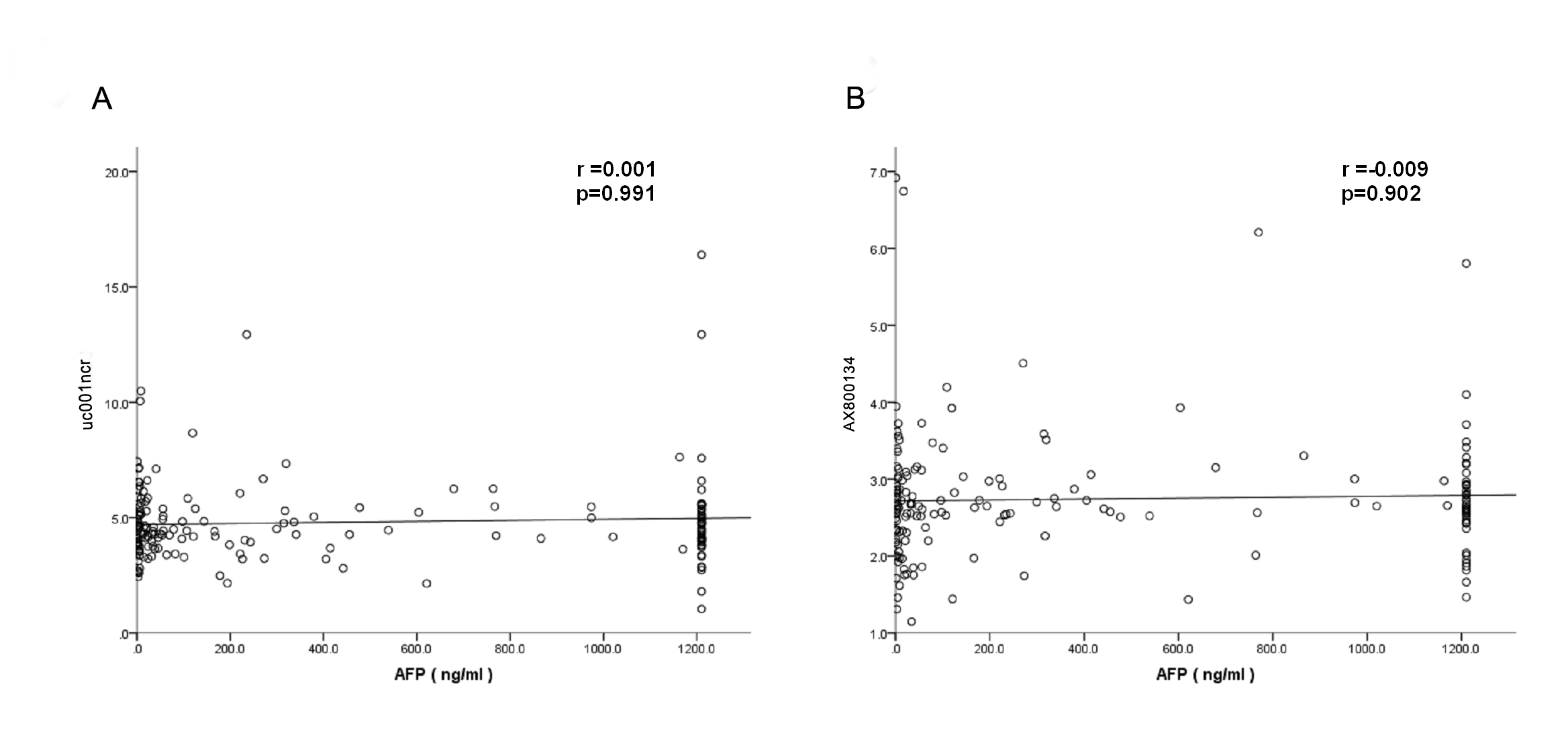

Supplement: S4 Fig — A for uc001ncr and B for AX800134. (TIF) [file pone.0144934.s004.tif]
