# Supplementary material for: Serum LncRNAs Profiles Serve as Novel Potential Biomarkers for the Diagnosis of HBV-Positive Hepatocellular Carcinoma
Source: PLoS One. 2015 Dec 16;10(12):e0144934. doi: 10.1371/journal.pone.0144934 (PMC4684503; doi:10.1371/journal.pone.0144934)
Supplement: S1 Table — (DOCX) [file pone.0144934.s005.docx]

**S1 Table. Eligibility Criteria for Selection of the Subjects.**

General inclusion criteria

1. Age ≥18 years and ≤90 years

2. Not currently residing in an institution, such as a prison, nursing home, or shelter

3. Not severely ill in the intensive care unit

4. With the capability to give informed consent

5. Encountered between August 2008 and June 2010

Healthy volunteers (N group)

1. Had the medical check-up in Eastern Hepatobiliary Surgery Hospital

2. In healthy condition without malignancy

Chronic hepatitis B patients† (CHB group)

1. HBsAg-positive >6 months

2. Serum HBV DNA >20,000 IU/mL (105copies/mL), lower values 2,000- 20,000 IU/mL (104-105copies/mL) are often seen in HBeAg-negative chronic hepatitis B

3. Persistent or intermittent elevation in ALT/AST levels

4. Liver biopsy showing chronic hepatitis with moderate or severe necro - inflammation. Do not meet the diagnosis criteria of cirrhosis

HBV-related HCC patients (HCC group)

1. With HBV infection

2. Diagnosed by two experienced pathologists

3. If no tissue available, diagnosis must be supported by two image reports (ultrasound B, CT or MRI) and/or AFP

4. No pre-operative chemotherapy, radiotherapy, transarterial chemoembolization or ablation

5. Undergoing curative resection(R_0_), defined as complete macroscopic removal of the tumor and resected margin was negative pathologically

6. HCC was [confirm](app:ds:confirm)ed histologically in the resected specimens.

†From AASLD Practice Guidelines (2009) and Zhou J, et al. Plasma microRNA panel to diagnose hepatitis B virus-related hepatocellular carcinoma. J Clin Oncol 2011;29:4781-4788.
