# Supplementary material for: Serum LncRNAs Profiles Serve as Novel Potential Biomarkers for the Diagnosis of HBV-Positive Hepatocellular Carcinoma
Source: PLoS One. 2015 Dec 16;10(12):e0144934. doi: 10.1371/journal.pone.0144934 (PMC4684503; doi:10.1371/journal.pone.0144934)
Supplement: S2 Table — (DOCX) [file pone.0144934.s006.docx]

**S2 Table. Primary Oligonucleotide Sequences Used in this Study.**

| Gene | | **Sequence** | | Product size (bp) |
| --- | --- | --- | --- | --- |
| GAPDH | Primer S | 5ˊ- CCGGGAAACTGTGGCGTGATGG -3′ | | 309 |
|  | Primer A | 5ˊ- AGGTGGAGGAGTGGGTGTCGCTGTT-3′ | | |
| AK128595 | Primer S | 5ˊ- TGTTCTCACACCCTCTTACCGT -3′ | 112 | |
|  | Primer A | 5ˊ- GACATCCACTCCGACCTCTTTA -3′ | | |
| AX800134 | Primer S | 5ˊ- TCCCACTCCTGATGTTGAACC-3′ | 263 | |
|  | Primer A | 5ˊ- GGTGGCAGCACAGGTTTTGA -3′ | | |
| uc009ycz | Primer S | GGCTGTTTTGTGATGCGTGT | 154 | |
|  | Primer A | GGAGTGAGGGGTGCAGTTAG | | |
| NR_027300 | Primer S | GTGTCTCGGACCCCTTTGAG | 158 | |
|  | Primer A | CTTGTGGGGGAACTCCACTC | | |
| uc001ncr | Primer S | 5ˊ- GAAGAAGGCGCGCTACAATG -3′ | 198 | |
|  | Primer A | 5ˊ- CCACTGGCCAGGACTCAAAA -3′ | | |
