# Supplementary material for: Serum LncRNAs Profiles Serve as Novel Potential Biomarkers for the Diagnosis of HBV-Positive Hepatocellular Carcinoma
Source: PLoS One. 2015 Dec 16;10(12):e0144934. doi: 10.1371/journal.pone.0144934 (PMC4684503; doi:10.1371/journal.pone.0144934)
Supplement: S3 Table — (DOCX) [file pone.0144934.s007.docx]

**S3 Table.Clinical Characteristics of 5 HCC Patients Used for LncRNA**

| Code | sex | Age(yr) | HBV | AFP  (µg/ml) |
| --- | --- | --- | --- | --- |
| YXZ | female | 63 | HBsAg+++；HBsAb+；HBeAg-；HBeAb+；HBcAb+；HBV-DNA6.9﹤10^5^ | 114.0 |
| MXH | male | 51 | HBsAg+++；HBsAb-；HBeAg+；HBeAb-；HBcAb+；HBV-DNA2.1 ×10^3^ | 1210.0 |
| BHH | male | 60 | HBsAg+++；HBsAb-；HBeAg+；HBeAb-；HBcAb+；HBV-DNA1.0×10^3^ | 1210.0 |
| HQW | male | 37 | HBsAg+++；HBsAb-；HBeAg+；HBeAb-；HBcAb+3.9×10^3^ | 2.3 |
| WXM | male | 48 | HBsAg+++；HBsAb-；HBeAg-；HBeAb+；HBcAb+；HBV-DNA1.0 ×10^3^ | 1210.0 |
