# Supplementary material for: Serum LncRNAs Profiles Serve as Novel Potential Biomarkers for the Diagnosis of HBV-Positive Hepatocellular Carcinoma
Source: PLoS One. 2015 Dec 16;10(12):e0144934. doi: 10.1371/journal.pone.0144934 (PMC4684503; doi:10.1371/journal.pone.0144934)
Supplement: S4 Table — (DOCX) [file pone.0144934.s008.docx]

**S4 Table. Expression Profiles of 5 LncRNAs on Microarrays**

| SEQ_ID | p-value | FCAb-  solute | regul-  ation | Chrom-  osome | seqname |
| --- | --- | --- | --- | --- | --- |
| AS000006138 | 4.15E-05 | 22.38713 | up | chr16 | AK128595 |
| AS000007482 | 2.55E-04 | 14.46045 | up | chr15 | AX800134 |
| AS000041409 | 1.72E-04 | 39.98358 | down | chr11 | uc009ycz |
| AS000033772 | 0.001859 | 36.70029 | down | chr19 | NR_027300 |
| AS000035890 | 8.00E-04 | 12.97875 | up | chr11 | uc001ncr |

FCAbsolute, the differential fold of gene expression between the tumor tissue and paired no

tumor tissue; Regulation, “up”/”down” means the genes was upregulation/downregulation the tumor tissue than in no tumor tissue;
