# Supplementary material for: Serum LncRNAs Profiles Serve as Novel Potential Biomarkers for the Diagnosis of HBV-Positive Hepatocellular Carcinoma
Source: PLoS One. 2015 Dec 16;10(12):e0144934. doi: 10.1371/journal.pone.0144934 (PMC4684503; doi:10.1371/journal.pone.0144934)
Supplement: S5 Table — (DOCX) [file pone.0144934.s009.docx]

**S5 Table.Clinical Characteristics of the HCC Patients.**

| Variables | | Cohotr1(Tissue) |  |  | | Cohort 2(Serum) | | |
| --- | --- | --- | --- | --- | --- | --- | --- | --- |
|  |  | HCC(68) | | HCC(n=50) | HBV(n=50) | | HC(n=50) |  |
| Age (years) | | | |  |  | |  |  |
| Mean±SD | 49.76±10.71 | | | 51.45±8.60 | 45.69±14.84 | | 51.18±6.0 |  |
| Sex |  | | |  |  | |  |  |
| Male/Female | 63/5 | | | 46/4 | 16/34 | | 41/9 |  |
| HbsAg |  | | |  |  | |  |  |
| Positive/Negative | 68/0 | | | 50/0 |  | |  |  |
| Total bilirubin (mmol/l) | | | | |  | |  |  |
| Mean±SD | 16.17±8.142 | | | 15.22±6.97 | 14.68±7.06 | | 13.14±5.44 |  |
| ALT (U/l) | | | |  |  | |  |  |
| Mean±SD | 48.37±27.35 | | | 43.71±26.27 | 25.48±14.26 | | 25.61±14.0 |  |
| Tumor size (cm) | | | |  |  | |  |  |
| Mean±SD | 10.39±4.305 | | | 6.09±4.03 |  | |  |  |
| Tumor number | | | |  |  | |  |  |
| Single/Multiple | | 44/24 | | 37/13 |  | |  |  |
| AFP(ng/ml) | | | |  |  | |  |  |
| Mean±SD | 552.2±541.3 | | | 355.1±494.2 | 2.5±2.07 | | 3.67±2.66 |  |
| Macrovascular invasion | | | |  |  | |  |  |
| Yes/No | | 34/34 | | 26/24 |  | |  |  |
| cirrhosis | | | |  |  | |  |  |
| Yes/No | | 50/18 | | 31/19 |  | |  |  |
| BCLC | |  | |  |  | |  |  |
| 0+A/B+C | | 38/30 | | 26/24 |  | |  |  |

Abbreviations: AFP, alpha fetoprotein; BCLC, Barcelona Clinic Liver Cancer；ALT, alanine aminotransferase; HCC, hepatocellular carcinoma；HBV, chronic hepatitis B; HC,Healthy Count
