# Supplementary material for: Serum LncRNAs Profiles Serve as Novel Potential Biomarkers for the Diagnosis of HBV-Positive Hepatocellular Carcinoma
Source: PLoS One. 2015 Dec 16;10(12):e0144934. doi: 10.1371/journal.pone.0144934 (PMC4684503; doi:10.1371/journal.pone.0144934)
Supplement: S6 Table — (DOCX) [file pone.0144934.s010.docx]

**S6 Table. Expression Profiles of 22 Candidate lncRNAs on qRT-PCR in 150 Samples.**

|  |  |  | Microarrays | | |  | | Detect in serum | |
| --- | --- | --- | --- | --- | --- | --- | --- | --- | --- |
| SEQ_ID | seqname | Chrom- | FCAb- | regul- | p-value | | rate(%) | | p-value |
|  |  | osome | solute | ation |  |  |  |  |  |
| AS000006138 | AK128595 | chr16 | 22.38713 | up | 4.15E-05 | | ND | | NA |
| AS000007482 | AX800134 | chr15 | 14.46045 | up | 2.55E-04 | | 100 | | <0.001 |
| AS000035890 | uc001ncr | chr11 | 12.97875 | up | 8.00E-04 | | 100 | | <0.001 |
| AS000004168 | AK095208 | chrY | 10.82225 | up | 0.001241 | | <50 | | NA |
| AS000041668 | uc010aoj | chr14 | 9.68315 | up | 1.59E-04 | | <50 | | NA |
| AS000004886 | AK123488 | chr5 | 8.836597 | up | 0.002794 | | 100 | | 0.2364 |
| AS000038692 | uc002zxz | chr22 | 7.738495 | up | 0.017476 | | 100 | | 0.3301 |
| AS000038691 | uc002zxy | chr22 | 7.664648 | up | 0.012828 | | ND | | NA |
| AS000039783 | uc003olt | chr6 | 7.240793 | up | 1.07E-04 | | ND | | NA |
| AS000032502 | NR_002935 | chr7 | 7.211474 | up | 4.13E-04 | | ND | | NA |
| AS000036035 | uc001pws | chr11 | 7.065984 | up | 1.84E-04 | | <50 | | 0.3022 |
| AS000041577 | uc009zrm | chr12 | 6.867964 | up | 0.005431 | | <50 | | 0.2361 |
| AS000000408 | AF085935 | chrX | 6.778407 | up | 7.50E-05 | | 73.3 | | <0.001 |
| AS000040316 | uc003wbd | chr7 | 6.664486 | up | 0.003756 | | 68.7 | | <0.001 |
| AS000001003 | AF147373 | chr8 | 6.61452 | up | 8.15E-04 | | 100 | | 0.44264 |
| AS000040321 | uc003wbn | chr7 | 6.312547 | up | 0.001777 | | 100 | | 0.1056 |
| AS000039721 | uc003nhn | chr6 | 6.206917 | up | 5.35E-04 | | ND | | NA |
| AS000011241 | HIV1921 | chr6 | 6.138757 | up | 4.48E-04 | | ND | | NA |
| AS000042934 | uc010mes | chr8 | 5.937569 | up | 9.52E-04 | | 100 | | 0.5761 |
| AS000042186 | uc010fha | chr2 | 5.67579 | up | 0.006032 | | 100 | | 0.3243 |
| AS000042682 | uc010jvl | chr6 | 5.508106 | up | 0.002427 | | ND | | NA |
| AS000040514 | uc003ybu | chr8 | 5.219843 | up | 0.006233 | | ND | | NA |
| AS000040308 | uc003war | chr7 | 5.133719 | up | 1.29E-04 | | <50 | | NA |

FCAbsolute, the differential fold of gene expression between the tumor tissue and paired no

tumor tissue; Regulation, “up”/”down” means the genes was upregulation/downregulation the tumor tissue than in no tumor tissue; ND,not determined, the lncRNAs did not pass the quality control process in which the lncRNAs showed CT values above 35 cycles in > 50% of the 150samples; NA, not application .
