# Supplementary material for: Serum LncRNAs Profiles Serve as Novel Potential Biomarkers for the Diagnosis of HBV-Positive Hepatocellular Carcinoma
Source: PLoS One. 2015 Dec 16;10(12):e0144934. doi: 10.1371/journal.pone.0144934 (PMC4684503; doi:10.1371/journal.pone.0144934)
Supplement: S1 Text — (DOCX) [file pone.0144934.s011.docx]

**S1 Text. Sample Preparation and the Methods**

For serum preparation, peripheral blood (5ml) was drawn into EDTA tubes. Within 30 minutes, the tubes was centrifuged at 1600 rpm for 5 min and the serum aliquoted into 1.5 ml Eppendorf tubes, followed by a 15 min high speed centrifugation at 12000 rpm to completely remove cell debris, leaving only circulating RNA. Subsequently, the supernatant was transferred to fresh tubes and stored them at –80 °C.

Total RNA was extracted from 300 μL of serum using a Blood Total RNA Isolation

Kit (RP4001, BioTeke, Beijing, China) and eluted in 30 μL of pre-heated (95˚C)

Elution Solution according to the manufacturer’s instructions. RNA concentration and purity was measured on a Nanodrop spectrophotometer (Thermo Scientific, Waltham, MA). The OD260/280 ratios for all samples were between 1.8 and 2.0.

RT and qPCR kits were used to evaluate the expression levels of the selected lncRNAs. RT reactions were performed in a volume of 30 μL using a PrimeScript® RT reagent Kit (Takara, Dalian, China) incubated for 15 min at 37°C and 5 s at 85°C, followed by storage at 4°C. For real-time PCR, 1 μL of diluted RT product was mixed with 10 μL of 2 × SYBR® Premix Ex TaqTM (Takara, Dalian, China), 0.6 μL of gene-specific forward and reverse primers (10 μM), and 8.4 μL of nuclease-free water in a final volume of 20 μL according to the manufacturer’s instructions. All reactions were performed using an Eppendorf Mastercycler EP Gradient S (Eppendorf, Germany) with the following conditions: 95°C for 30 s, followed by 40 cycles of 95°C for 5 s and 60°C for 30 s. Samples were analyzed in triplicate and included no-template controls. Amplification of the appropriate product was confirmed by melting curve analysis following amplification.

The relative expression of each lncRNA was calculated using the comparative cycle threshold (CT) (2^−△△CT^) method with GAPDH as the endogenous control for data normalization. The CT was defined as the number of cycles required for the SYBR signal to cross the threshold. Samples with a CT > 40 were considered negative. △CT was calculated by subtracting the CT values of GAPDH from the CT values of the chosen lncRNA. △△CT was then calculated by subtracting the mean △CT of the healthy control samples from the △CT of the HCC or HBV samples. The fold change of the lncRNA was calculated using the equation 2^−△△CT^.
